# Comparing upfront surgery with neoadjuvant treatments in patients with resectable, borderline resectable or locally advanced pancreatic cancer: a systematic review and network meta-analysis of randomized clinical trials

**DOI:** 10.1097/JS9.0000000000001313

**Published:** 2024-03-18

**Authors:** Jiaxin He, Na Lv, Zhenyi Yang, Yixuan Luo, Wen Zhong, Chunli Wu

**Affiliations:** Department of Radiation Oncology, The Fourth Affiliated Hospital of China Medical University, Liaoning, China

**Keywords:** neoadjuvant, network meta-analysis, pancreatic cancer, upfront surgery

## Abstract

**Background:**

The aim was to explore the optimal neoadjuvant therapy strategy for resectable, borderline resectable, and locally advanced pancreatic cancer, in order to provide a theoretical basis for the development of new neoadjuvant treatment protocols for clinical use.

**Patients and methods:**

The authors reviewed literature titles and abstracts comparing three treatment strategies (neoadjuvant chemoradiotherapy, neoadjuvant chemotherapy, and upfront surgery) in PubMed, Embase, The Cochrane Library, Web of Science from 2009 to 2023 to estimate relative odds ratios for resection rate and hazard ratios (HRs) for overall survival (OS) in all include trials.

**Results:**

A total of nine studies involving 889 patients were included in the analysis. The treatment methods included upfront surgery, neoadjuvant chemotherapy, and neoadjuvant chemoradiotherapy followed by surgery. The network meta-analysis results demonstrated that neoadjuvant chemoradiotherapy followed by surgery was an effective approach in improving OS for resectable and borderline resectable pancreatic cancer (RPC) patients compared to upfront surgery (HR: 0.79, 95% CI: 0.64–0.98) and neoadjuvant chemotherapy (HR: 0.79, 95% CI: 0.64–0.98). Additionally, neoadjuvant chemoradiotherapy significantly increased the margin negative resection (R0) rate and pathological negative lymph node (pN0) rate in patients with resectable and borderline RPC. However, it is worth noting that neoadjuvant chemoradiotherapy increased the risk of grade 3 or higher treatment-related adverse events, including in patients with locally advanced pancreatic cancer.

**Conclusions:**

The current evidence suggests that neoadjuvant chemoradiotherapy followed by surgery is the optimal choice for treating patients with resectable and borderline RPC. Future research should focus on optimizing neoadjuvant chemoradiotherapy regimens to effectively improve OS while reducing the occurrence of adverse events.

## Introduction

Highlights

- Exploring the optimal neoadjuvant therapy strategy for resectable, borderline resectable, and locally advanced pancreatic cancer.
- Neoadjuvant chemoradiotherapy is effective in improving overall survival for resectable and borderline resectable pancreatic cancer.
- Neoadjuvant chemoradiotherapy also increases the margin negative resection rate and pathological negative lymph node rate.
- However, neoadjuvant chemoradiotherapy increases the risk of grade 3 or higher treatment-related adverse events.
- Further research is needed to optimize neoadjuvant therapy, balancing survival outcomes and reducing adverse events.

Pancreatic cancer is known for its poor overall prognosis and low resectability rates^1^. Despite tumor removal, long-term survival remains limited. Based on the extent of venous and arterial vascular involvement, nonmetastatic pancreatic cancer can be categorized into three groups: resectable, borderline resectable, and locally advanced^2^. Complete surgical resection at an early stage is the sole curative option for pancreatic cancer, as such, adjuvant therapy after surgery has become the standard treatment for resectable pancreatic cancer (RPC). Unfortunately, owing to a lack of early clinical symptoms and efficient screening modalities, most patients suffer from locally advanced or metastatic cancer during their initial presentation. Consequently, it is estimated that only 20% of newly diagnosed patients are suitable for resection without undergoing neoadjuvant treatment^3^. Yet, even in this most favorable cohort with RPC, up to 80% of patients recur after a short recurrence-free interval^4–6^. This high rate of recurrence has been attributed to the presence of occult micrometastatic disease at the time of resection and the lack of effective systemic therapies^7,8^, indicating that surgery alone is typically inadequate.

Pancreatic cancer, similar to breast cancer, should be considered as a systemic disease^9^ that necessitates systemic control. Neoadjuvant chemotherapy (radiotherapy) has been proposed as a new therapeutic strategy to convert unresectable tumors into resectable ones and/or to improve the resection rate of negative margins and reduce the incidence of lymph node metastasis. First, current guidelines for locally advanced pancreatic cancer (LAPC) recommend a multidisciplinary approach for nonsurgical management in well-performed patients. A meta-analysis comprising 19 trials found that unresectable diseases, including borderline resectable pancreatic cancer (BRPC) and LAPC achieved comparable survival outcomes to RPC after neoadjuvant therapy^10^. Secondly, the most recent National Comprehensive Cancer Network Guidelines recommend the utilization of neoadjuvant chemoradiotherapy for BRPC, although these recommendations lack high-quality evidence^11,12^. In the real-world setting, the application of neoadjuvant chemoradiotherapy, particularly in RPC, remains controversial. Despite a few randomized controlled trials (RCTs) indicating improved survival rates in RPC or BRPC patients with the use of neoadjuvant chemoradiotherapy, these trials are limited by small sample sizes^13,14^. Thus, it is still necessary to aggregate existing research and conduct a comprehensive meta-analysis. Furthermore, to the best of our knowledge, although several meta-analyses have investigated the effect of neoadjuvant therapy compared with upfront surgery on nonmetastatic pancreatic cancer and concluded its certain advantages^15–18^, there is limited literature that has comprehensively compared the neoadjuvant chemoradiotherapy and neoadjuvant chemotherapy. For example, van Dam *et al.* ^16^ conducted a meta-analysis comparing the overall survival (OS) of the two neoadjuvant treatments with surgery, and ultimately found both to be superior to surgery. However, they were unable to determine the relative superiority of the two neoadjuvant treatments, Therefore, further research in this area is warranted.

This study aims to conduct a network meta-analysis (NMA) of RCTs evaluating the survival outcomes and resection of neoadjuvant chemoradiotherapy, neoadjuvant chemotherapy, and upfront surgery in patients with RPC, BRPC, and LAPC. The goal is to provide valuable insights into selecting appropriate treatment options.

## Patients and Methods

### Protocol registration

This NMA was registered in PROSPERO and the unique identifying number of the study was CRD42023409228. Moreover, this work has been reported in line with PRISMA, Supplemental Digital Content 1, http://links.lww.com/JS9/C123, Supplemental Digital Content 2, http://links.lww.com/JS9/C124 (Preferred Reporting Items for Systematic Reviews and Meta-Analyses)^19^ and AMSTAR, Supplemental Digital Content 3, http://links.lww.com/JS9/C125 (Assessing the methodological quality of systematic reviews) Guidelines^20^.

### Data sources and search

PubMed, Embase, Cochrane Database, and Web of Science were searched using the search strategy from January 1, 2009, which was the date of introducing the anatomical subtypes of pancreatic ductal adenocarcinoma, namely RPC, BRPC, and LAPC^21,22^, to September 1, 2023. The search strategy in PubMed included the following domains of Medical Subject Heading terms: “pancreatic neoplasms” and “neoadjuvant Therapy”. These terms were combined with “AND” or “OR” within the limitations of RCTs, which were provided in Supplementary Figure 1, Supplemental Digital Content 4, http://links.lww.com/JS9/C126. Additionally, relevant reviews and references of the included trials were manually searched to identify any additional relevant references.

### Study selection

We included trials that met the following criteria:

1. Participants: patients conformed to the diagnostic criteria of RPC, BRPC, or LAPC.
2. Compare two of the three treatment strategies: neoadjuvant chemoradiotherapy, neoadjuvant chemotherapy, and upfront surgery.
3. Reporting at least one of the following clinical outcomes: OS, resection rate, margin negative resection (R0) rate, pathological negative lymph node (pN0) rate, and incidence of Grade ≥3 treatment-related adverse events.
4. Study type: RCTs.

### Data extraction and risk of assessment

The study period, institution, and the number of participants, as well as information on treatments and protocols such as intervention and control regimens, along with outcome measures, were gathered and documented in a spreadsheet. Two independent authors conducted article evaluations and data extraction to avoid potential evaluation bias, while a third researcher resolved any discrepancies. The quality and risk of bias of RCTs were subsequently assessed using the Cochrane Risk of Bias Tool 2^23^, including random sequence generation, allocation sequence concealment, blinding, incomplete outcome data, selective publication, and other sources of bias.

### Outcomes

We integrated all relevant evidence in a Bayesian framework, such as hazard ratios (HRs) with corresponding 95% CI for survival outcomes and odds ratios (OR) for binary outcomes. The primary outcome was OS, while the secondary outcomes were the Resection rate, R0 resection rate, pN0 rate, and Grade greater than or equal to 3 treatment-related adverse events rate. R0 was defined as no tumor invasion within 1 mm of the surgical margin, and Grade greater than or equal to 3 treatment-related adverse events rate typically refers to the Common Terminology Criteria for Adverse Events (CTCAE) scale, which is a standard system used to grade the severity of adverse events that occur during cancer treatment. Grade 3 adverse events are considered severe, leading to significant interference with daily activities and may require medical intervention. For the primary outcome, HRs were extracted from the articles in which these data were provided. If the HRs were not reported, using indirect methods based on the survival curve provided in the article with Engauge Digitizer 4.1 software (Markmitch, Boston, Massachusetts)^24^.

### Data synthesis and analysis

Statistical analyses were performed using STATA version 16.0 software. *Q* test and *I*^2^ statistic were used to evaluate heterogeneity across the involved studies. When *I*^2^ less than 50% and *P* greater than 0.10, the fixed effect model was used to combine data sets. Otherwise, the random effect model was applied^25^. For the HR and 95% CI of the outcome indicators of the OS. Bayesian NMA was performed using the JAGS and GEMTC packages in R.3.6.1 and Markov chain Monte Carlo simulation technology^26^ with 50 000 sample iterations, 50 000 burn-in cycles, and a thinning interval of 10^27^. Hypotheses were assessed using a node-splitting method, which assesses the difference between indirect and direct evidence, and a consistency model was used when *P* greater than 0.05. We used the Surface Under the Cumulative Ranking (SUCRA) curve to assess the likelihood of each treatment method being ranked as the top choice, with higher SUCRA values indicating greater probability^28^. Sensitivity analysis was conducted to explore the source of heterogeneity, and publication bias was assessed using a funnel plot.

## Result

### Eligible studies and characteristics

**Figure 1:**
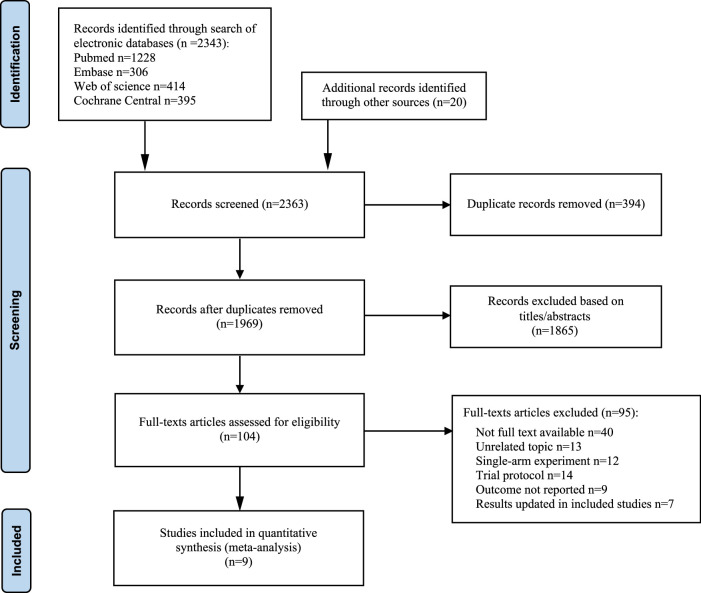
Flow diagram of literature search and selection process.

A total of 2363 studies were initially identified from electronic databases (PubMed: 1228; Embase: 306; Cochrane Database: 395; Web of Science: 414; Other sources: 20). After removing duplicates and irrelevant studies based on title and abstract (Including topics related to literature reviews, conferences, letters, animal experiments, and content inconsistencies), 104 studies underwent full-text review, and 9 RCTs^13,14,29–35^ were included in the final data synthesis (Fig. 1), one of these studies was a four-arm study.

The characteristics of the included studies are summarized in Table 1. Of these nine studies, four included patients with RPC disease, three with BRPC disease, and two included RPC/BRPC and LAPC tumors, respectively. Table 2 summarizes the characteristics of patients who underwent chemoradiotherapy, chemotherapy, and upfront surgery. Of the 889 patients, 303 (34%) were assigned to neoadjuvant chemoradiotherapy, 237 (26.7%) were allocated to neoadjuvant chemotherapy, and 349 (39.3%) received upfront surgery. Among them, six studies^13,14,29,31–33^ reported the proportion of patients receiving adjuvant chemotherapy, with 142 out of 208 patients (68.3%) undergoing upfront surgery, 64 out of 141 patients (45.4%) in the neoadjuvant chemotherapy group, and 96 out of 251 patients (38.2%) in the neoadjuvant chemoradiotherapy group. Two studies further reported the number of patients who completed adjuvant chemotherapy, with 42 out of 69 patients in the neoadjuvant chemoradiotherapy group and 41 out of 78 patients in the upfront surgery group, while no reporting was available for the neoadjuvant chemotherapy group.

**Table 1:** Demographics of included studies

References	Institution	Country	Period of study	median follow-up(m)	Tumor	Definition of status	Patients (n)	Male (%)	Median age (y)
Casadei *et al.* ^29^	Single-	Italy	2003–2009	40	PR	<180 SMV/PV; No CA/HA/SMA contact	38	57.9	70 (48–79)
Reni *et al.* ^14^	Multi-	Italy	2010–2015	54.5	PR	No invasion of SMA/SMV/PV/CA/HA	88	59	66 (37–75)
Versteijne*et al.* ^35^	Multi-	Netherlands	2013–2017	27	PR/BR	PR:<90 SMV/PV; No CA/HA/SMA contact BR:<90CA/HA/SMA; No 90–270 PV/SMV contact	246	56	67 (59–73)
Loehrer *et al.* ^34^	Single-	American	2003–2005	N/A	LAPC	Cytologic or histologic evidence of locally unresectable, or not complete resection based on clinical or radiographic	71	52.1	66.2 (46.9–83.7)
Jang *et al.* ^13^	Multi-	Korea	2012–2014	24	BR	2012 NCCN criteria	50	64	59
Golcher *et al.* ^32^	Multi-	Germany	2003–2009	61	PR	≤180 HA/SMA/CA	66	53	63.9 (33–76)
Katz *et al.* ^33^	Multi-	American	2016–2019	43	BR	NA	126	51	64 (37–83)
Ettrich *et al.* ^30^	Multi-	Germany	2015–2019	NA	PR	No CA/HA/SMA contact	118	56.3	56
Ghaneh *et al.* ^31^	Multi-	United Kingdom	2014–2018	12.2	BR	2013 NCCN criteria	86	44	63.5

BR, borderline resectable; CA, celiac axis; HA, hepatic artery; NA, not available; NCCN, National Comprehensive Cancer Network; PR, potentially resectable; PV, portal vein; SMA, superior mesenteric artery; SMV, superior mesenteric vein.

**Table 2:** Characteristics of the randomized controlled trials included in the meta-analysis

References	Intervention	Control	Regimen	Participants	Median OS	Resection rate (%)	R0 resection rate (%)	pN0 rate (%)	Grade ≥3 treatment-related adverse events (%)
Casadei *et al.* ^29^	ChemoRT	US	Gem+RT (45 Gy)	18 vs. 20	22.4 vs. 19.5	61.1 vs. 75	63.6 vs. 33.3	45.5 vs. 13.3	38.9 vs. NA
Reni *et al.* ^14^	Chemo	US	Gem/Cis/Epi/Cap	32 vs. 56	38.2 vs. (20.4–26.4)	84.4 vs. 87.5	63 vs. 32.7	48.1 vs. 26.5	11.1 vs. 20.4
Versteijne *et al.* ^35^	ChemoRT	US	Gem+RT (36 Gy)	119 vs. 127	15.7 vs. 14.3	60.5 vs. 72.4	72.1 vs. 42.7	64.7 vs. 18.3	NA
Loehrer *et al.* ^34^	ChemoRT	Chemo	Gem+RT (50.4 Gy) and Gem	34 vs. 37	11.1 vs. 9.2	NA	NA	NA	82.4 vs. 80
Jang *et al.* ^13^	ChemoRT	US	Gem+RT (45 Gy)	27 vs. 23	22.4 vs. 19.5	63 vs. 78.3	82.4 vs. 33.3	70.6 vs. 16.7	11.1 vs. 4.3
Golcher *et al.* ^32^	ChemoRT	US	Gem, Cis+ RT (55.8 Gy)	33 vs. 33	25 vs. 18.9	57.6 vs. 69.7	89.5 vs. 69.6	68.4 vs. 43.5	26.3 vs. 47.8
Katz *et al.* ^33^	ChemoRT	Chemo	mFOLFIRINOX+RT(33–40 Gy) vs. mFOLFIRINOX	56 vs. 70	17.1 vs. 29.8	33.9 vs. 45.7	73.7 vs. 87.5	47.4 vs. 46.9	63.6 vs. 56.9
Ettrich *et al.* ^30^	Chemo	US	Gem+PTX	59 vs. 59	25.2 vs. 16.7	69.5 vs. 78	87.8 vs. 67.4	33.3 vs. 29.5	NA
	Chemo	US	Gem/Cap	39 vs. 31		56.4 vs. 67.7	18.2 vs. 14.3	31.8 vs. 9.5	36.1 vs. 7.1
Ghaneh *et al.* ^31^	ChemoRT	US	Cap+RT (50.4 Gy)	16 vs. 31	NA	50 vs. 67.7	37.5 vs. 14.3	25 vs. 9.5	28.6 vs. 7.1
	ChemoRT	Chemo	Cap+RT (50.4 Gy) vs. Gem/Cap	16 vs. 39		50 vs. 56.4	37.5 vs. 18.2	25 vs. 31.8	28.6 vs. 36.1

Cap, capecitabine; Cis, cisplatin; Epi, epirubicin; Gem, gemcitabine; NA, not available; PTX, Paclitaxel; US, upfront surgery.

Baseline information was available in eight articles^13,14,29,31–35^, including a total of 771 patients. Among them, the proportion of male patients was 50.3% (49.5% in the neoadjuvant chemoradiotherapy group, 53.4% in the neoadjuvant chemotherapy group, and 49.3% in the upfront surgery group). There was a total of 299 patients with pancreatic head cancer, accounting for 38.8% of the total (21.8% in the neoadjuvant chemoradiotherapy group, 30% in the neoadjuvant chemotherapy group, and 62.1% in the upfront surgery group). The final combined rates for neoadjuvant chemoradiotherapy, neoadjuvant chemotherapy, and upfront surgery were as follows: resection rate of 54.4, 64.45, and 75.5%, R0 resection rate of 69.8, 64.1, and 41.9%, pN0 rate of 53.6, 40, and 22.5%, and grade greater than or equal to 3 treatment-related adverse events of 41.8, 46.1, and 19.9%.

Overall, all studies used at least chemotherapy as neoadjuvant treatment, the common regimens included Gemcitabine (eight studies, N=540), and modified FOLFIRINOX (mFOLFORINOX) in one study (N=126). Gemcitabine was also the main adjuvant chemotherapy regimen in almost all studies. Seven studies reported specific radiotherapy regimens and doses, no study used radiotherapy as the sole neoadjuvant treatment, total administered dose ranged from 25 to 50.4 Gy. One of these studies included 40 patients (73%) who received stereotactic body radiation therapy (SBRT) [35 (87.5%)] or hypofractionated image-guided radiation therapy [5 (12.5)], treatment with SBRT comprised 33–40 Gy in five fractions, and hypofractionated image-guided radiation therapy comprised 25 Gy in five fractions.

A common risk of bias in these studies is the within-study and between-study heterogeneity of neoadjuvant therapy (Supplementary Fig. 2, Supplemental Digital Content 5, http://links.lww.com/JS9/C127; Supplementary Fig.3, Supplemental Digital Content 6, http://links.lww.com/JS9/C128). In addition, the duration of follow-up varied widely among the studies, with a minimum of 12.2 months and a maximum of 61 months in known data. Furthermore, different criteria were used for resectability. One study decided on early termination based on the statistical significance effect of neoadjuvant therapy.

### Survival analysis

**Figure 2:**
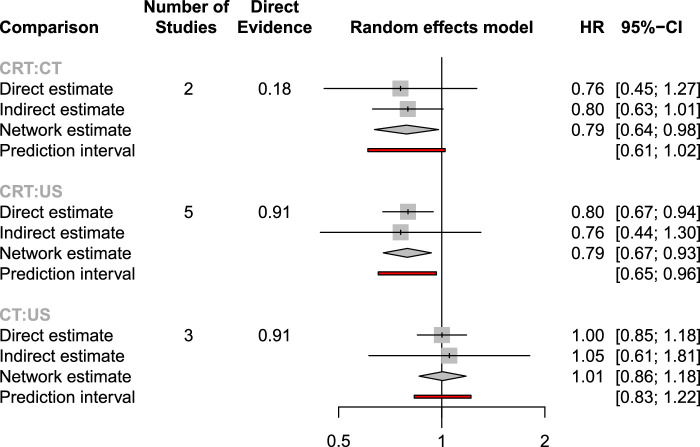
Node-splitting method in comparison between direct and indirect evidence of overall survival (OS). CRT, chemoradiotherapy; CT, chemotherapy; HR, hazard ratio; US, upfront surgery.

Eight studies (including only PRC/BRPC)^13,14,29,31–33,35^ involving 803 patients were assessed in the comparison of OS (Fig. 2), neoadjuvant chemoradiotherapy prolonged the OS of patients significantly compared with neoadjuvant chemotherapy (HR: 0.79, 95% CI: 0.64–0.98) and upfront surgery (HR: 0.79, 95% CI: 0.67–0.93). The SUCRA value of chemoradiotherapy (0.99) was the largest for OS, including its likelihood of being ranked first, followed by upfront surgery (0.26) and neoadjuvant chemotherapy (0.24) (Supplementary Fig. 4, Supplemental Digital Content 7, http://links.lww.com/JS9/C129).

### Resection rate

**Figure 3:**
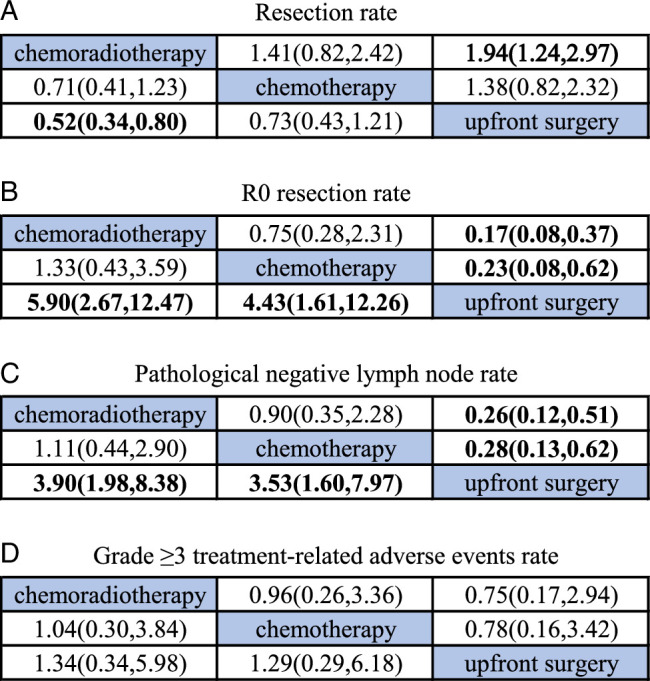
Odds ratios and 95% CIs for three treatment comparisons: (A) Resection rate, (B) R0 resection rate, (C) pathological negative lymph node rate, (D) grade greater than or equal to 3 treatment-related adverse events rate.

**Figure 4:**
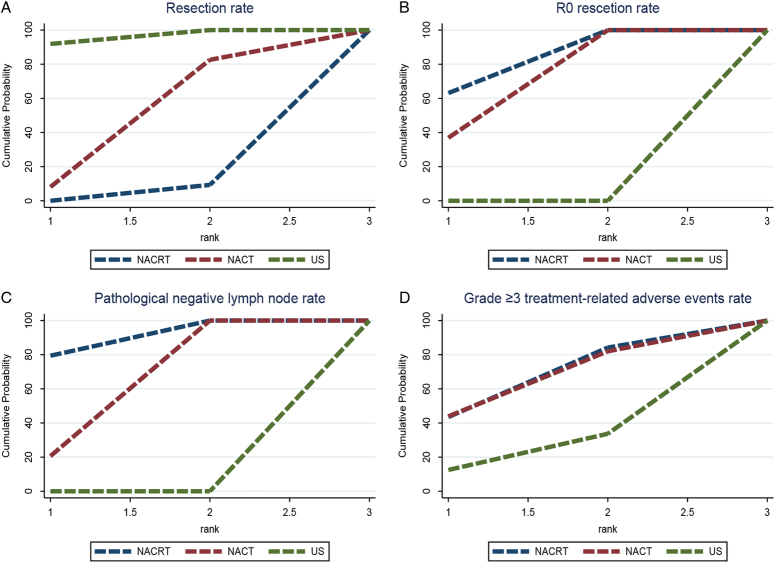
The cumulative ranking plot with the surface under the cumulative ranking curve (SUCRA) for the outcome: (A) resection rate, (B) R0 resection rate, (C) pathological negative lymph node rate, (D) grade greater than or equal to 3 treatment-related adverse events rate. NACRT, neoadjuvant chemoradiotherapy; NACT, neoadjuvant chemotherapy; US, upfront surgery.

A total of eight RCTs^13,14,29–33,35^ were summarized and combined, and the results indicated that neoadjuvant chemoradiotherapy showed a significantly lower resection rate over upfront surgery (Fig. 3A, OR: 0.52, 95% CI: 0.34–0.80), and neoadjuvant chemotherapy did not show superior efficacy in resection rate when compared to upfront surgery (OR: 0.71, 95% CI: 0.41–1.23). Furthermore, the SUCRA revealed that upfront surgery accounted for a higher probability of success (Fig. 4A) compared to neoadjuvant chemotherapy and neoadjuvant chemoradiotherapy.

### R0 resection rate

By utilizing an NMA approach, we conducted a comparative analysis of the effects of these different therapies on the R0 resection rate. Our study findings indicate that neoadjuvant chemoradiotherapy was more effective in achieving the R0 resection rate in comparison to upfront surgery alone (Fig. 3B, OR: 5.90, 95% CI: 2.67–12.47), as depicted in Fig. 4B. Additionally, neoadjuvant chemotherapy considerably increased the R0 resection rate compared to upfront surgery (OR: 4.43, 95% CI: 1.61–12.26).

### Pathological negative lymph node rate

Seven out of the nine studies^13,14,29,31–33,35^ included in the analysis reported the number of people with pathological negative lymph nodes. Our integrated data analysis revealed that the probability of achieving pN0 was significantly higher with neoadjuvant chemoradiotherapy compared to upfront surgery alone (Fig. 3C, OR: 3.90, 95% CI: 1.98–8.38). Similarly, the probability of achieving pN0 was also higher with neoadjuvant chemotherapy compared to upfront surgery (OR: 3.53, 95% CI: 1.60–7.97). And the SUCRA of neoadjuvant chemoradiotherapy was higher than neoadjuvant chemotherapy (Fig. 4C).

### Grade ≥3 treatment-related adverse events rate

A multitude of grade greater than or equal to 3 treatment-related adverse events were reported in seven studies^13,14,29,31–34^, with gastrointestinal toxicity (nausea, vomiting, and diarrhea), hematological abnormalities (neutropenia, anemia, and thrombocytopenia), and infection being the most prevalent. Notably, Loehrer *et al.* ^34^ found that while grade 3 and 4 toxicities were comparable between the chemoradiotherapy and chemotherapy groups (79% vs. 77%), grade 4 and 5 toxicities were considerably higher in the former (41%) than the latter (9%), with each group experiencing one grade 5 toxicity event (acute respiratory distress syndrome and cardiac ischemia). Despite no statistically significant association between adverse events and treatment choice (Fig. 3D), the risk was numerically highest with neoadjuvant chemoradiotherapy and lower with upfront surgery (Fig. 4D). Katz *et al.* ^33^ reported a grade 3 or higher toxicity rate of 64% in the chemoradiotherapy group in a trial comparing preoperative mFOLFIRINOX plus hypofractionated radiotherapy to mFOLFIRINOX alone.

## Discussion

Our study builds upon previous research by incorporating recent and high-quality RCTs with a larger sample size, thus enabling more precise conclusions to be drawn. We demonstrate that neoadjuvant chemoradiotherapy might prolonged the OS of PRC and BRPC. Upon conducting indirect comparisons to explore secondary outcomes, our findings are consistent with previous meta-analyses, indicating that patients except for LAPC who received neoadjuvant therapy have lower resection rates than those who underwent upfront surgery. This phenomenon may be attributed to several factors. First, neoadjuvant therapy may not effectively control the tumor in all tumors, as not all tumors may be reduced or eliminated^33,36^. Second, neoadjuvant therapy may cause adverse effects on the patient’s body, with chemotherapy and radiotherapy potentially resulting in a range of toxicities, such as nausea, vomiting, fatigue, and hair loss, which may impact the patient’s physical performance and increase the difficulty or risk of surgery^37^. Third, some patients may be unsuitable for surgery due to the presence of other health problems that may contribute to a lower surgical resection rate, even if the tumor is controlled after neoadjuvant therapy. Furthermore, the R0 resection rate and pN0 rate are important factors that impact patient survival, and neoadjuvant therapy is superior to upfront surgery in this regard, which supports the notion that neoadjuvant therapy leads to higher OS. However, it is important to note that the definition of R0 resection may vary between studies^11^, and this can potentially affect the final statistical analysis. Interestingly, the SUCRA of neoadjuvant chemoradiotherapy was the largest in R0 resection rate and pN0 rate, but also had the highest risk of grade 3 and above treatment-related adverse events, however, given that this complication encompassed patients diagnosed with LAPC, it is essential to exercise caution when interpreting its influence on the outcomes. This highlights the importance of weighing the potential advantages and drawbacks of different neoadjuvant strategies in pancreatic cancer management and underscores the need for a personalized treatment approach tailored to the specifics of each patient’s case. Further studies are needed to provide more conclusive evidence and develop innovative strategies to optimize neoadjuvant therapy for pancreatic cancer patients.

The initial application of neoadjuvant therapy was primarily aimed at patients with advanced or locally advanced breast cancer who were not eligible for surgery^38^. In contrast, in other tumors, particularly pancreatic cancer, neoadjuvant therapy is generally considered to be mainly used for RPC or BRPC, while the term “conversion therapy” is more applicable to some patients with locally advanced or metastatic cancer. However, with advancements in surgical techniques, the two treatment strategies are now occasionally combined to achieve better treatment outcomes. Furthermore, patients who undergo upfront surgical treatment often receive additional adjuvant therapy at a later stage to further improve their chances of survival. Nevertheless, a minority of patients may refuse or prematurely terminate adjuvant therapy due to various reasons, which might affect the reliability of the associated findings. Therefore, this investigation represents a novel NMA evaluating the survival outcomes and feasibility of neoadjuvant therapy in patients with RPC, BRPC, and LAPC. While Hu *et al.* ^39^ have previously examined this topic, its scope was limited to only retrospective studies, including 14 studies, and focused solely on OS and R0 resection rates. A direct comparison between the two neoadjuvant treatments revealed superior OS with neoadjuvant chemoradiotherapy. The NMA, which indirectly compares the treatments, did not reach the same conclusion regarding OS and only demonstrated a significant difference in terms of R0 resection rates. Additionally, it identified a potential association between neoadjuvant chemotherapy and increased postoperative complications.

For pancreatic cancer, an outstanding concern is the use of neoadjuvant radiation therapy in addition to neoadjuvant chemotherapy. Previous studies have investigated neoadjuvant radiotherapy using conventional or hypofractionated radiation, but the protocols used in these studies were inconsistent, and stereotactic body radiation therapy was not applied in most of them. The present study^40^ utilized data from the National Cancer Data Base database and analyzed the survival outcomes of RPC patients who received preoperative chemotherapy alone, preoperative concurrent chemoradiotherapy, and preoperative SBRT with chemotherapy between 2010 and 2015. The results showed that patients treated with SBRT with chemotherapy had significantly improved survival compared to preoperative chemotherapy alone (30 vs. 21 months, *P*=0.02) and concurrent chemoradiotherapy (29 vs. 16 months, *P*=0.002). Recently, a phase II trial (Alliance A021501)^33^ has investigated the use of SBRT as a neoadjuvant option for BRPC. However, there is still a lack of well-studied evidence for the effectiveness of SBRT as neoadjuvant therapy in this patient population. The A021501 trial randomly assigned patients with BRPC to either chemoradiotherapy or chemotherapy alone, and the results showed an OS of 17.1 months versus 29.8 months, a resection rate of 33.9 versus 45.7%, and a rate of grade 3 or higher adverse events of 64 versus 57%, respectively. Based on these findings, the chemotherapy group was considered to be more effective, and the use of chemoradiotherapy was rejected. Also in the CONKO 007 phase III trial^41^, patients with LAPC were allocated to receive induction chemotherapy followed by chemoradiotherapy or chemotherapy. The study found that while OS rates did not significantly differ between the two groups, the chemoradiotherapy group experienced significantly higher rates of grade 3 or higher adverse events compared to the chemotherapy group (70 vs. 40%). These results suggest that the efficacy and toxicity of radiotherapy are dependent on the specific subtype and dose of treatment administered and that the addition of radiotherapy may not always be the best choice for treating all pancreatic cancer. To determine the ideal subtype and dose of radiotherapy for treating pancreatic cancer, further prospective randomized evaluations are needed. It is also important to acknowledge the heterogeneity of radiotherapy available for treating pancreatic cancer, which can significantly impact OS rates in patients.

Retrospectively, neoadjuvant therapy has emerged as a preferred treatment option for numerous gastrointestinal malignancies, and extensive clinical investigations have focused on refining this therapeutic modality. Rectal adenocarcinoma serves as an exemplary model, as several phase III trials have explored the optimal neoadjuvant therapy regimens^42,43^. However, there is an imperative need to shift our attention towards pancreatic cancer and to gain a better understanding of how to optimize neoadjuvant therapy for this malignancy. This entails identifying the most effective combination of chemotherapeutic agents, determining the appropriate duration of therapy, evaluating the usefulness of different radiation therapy strategies, exploring the utility of biomarkers such as CA19-9 or molecular subtypes in guiding neoadjuvant therapy, whether it can be used as a predictive biomarker for determining the ideal neoadjuvant treatment strategy, and the optimal timing of surgery after neoadjuvant therapy. In particular, the timing of surgery remains a subject of exploration in the management of pancreatic cancer. Following neoadjuvant chemoradiotherapy, surgical resection is typically performed 4–8 weeks after radiotherapy completion. However, the optimal timing of surgery after neoadjuvant chemoradiotherapy has not been well established in BRPC and LAPC patients. Lin *et al.* ^44^ suggested that performing surgery within 6 weeks after completing SBRT might be associated with improved local control, independent of other prognostic factors. However, this timing also carries potential complications such as anatomic disruption or fibrosis that pose greater surgical challenges. While the benefits of early surgery after SBRT are promising, additional research is necessary to thoroughly assess both the advantages and potential drawbacks of this timing strategy, and to determine if it is appropriate for all patients. An informed and collective approach involving collaboration between multidisciplinary teams of radiation oncologists and surgeons is needed to optimize the timing and maximize the benefits of neoadjuvant therapy and surgery in pancreatic cancer management.

To summarize, it is important to acknowledge the limitations of this study when interpreting the results. First, the number of studies included in the analysis was relatively small, and the sample sizes of these studies were modest. Consequently, it was not possible to perform subgroup analyses to compare the effectiveness of different neoadjuvant treatment regimens for pancreatic cancer, particularly in determining which types of patients are more suitable for receiving neoadjuvant chemoradiotherapy and whether changes in their CA19-9 levels have prognostic value. Second, the heterogeneity of the neoadjuvant treatment protocols utilized in the studies precluded meaningful subgroup analyses. Third, some studies had to be terminated prematurely due to cumulative patient adverse events, and the ultimate OS outcomes failed to exhibit statistically significant differences. Fourth, the majority of the included studies have focused on gemcitabine as single-agent neoadjuvant chemotherapy, while contemporary practice favors multiagent chemotherapy regimens. Some research is already underway to explore the advantages and disadvantages of mFOLFIRINOX as a neoadjuvant treatment measure (such as NCT04927780, NCT03991962, NCT02128100), but the results have not been published. The Alliance A02150 is the only RCT which showed a benefit of mFOLFIRINOX over additional radiation. Therefore, future research should evaluate the efficacy and safety of different neoadjuvant therapy strategies for pancreatic cancer. This would enable further investigations into the most effective neoadjuvant treatment protocols for different stages of pancreatic cancer. Clinicians also need to consider the limitations of the existing literature when making therapeutic decisions and individualize treatment plans based on the unique characteristics of each patient to achieve the optimal therapeutic outcome. In this article, although the concept of what neoadjuvant therapy should be offered to RPC is hard to determine, patients with T1 and T2 lesions without evidence for distant metastasis or lymph node involvement do not usually receive neoadjuvant treatments. However, based on existing research results^45^, it can be suggested that if these patients have elevated serum Ca19-9 levels, they should be recommended to receive neoadjuvant therapy. As a result, according to findings from this meta-analysis, these treatments might include neoadjuvant chemoradiotherapy, even for T1/T2 N0 lesions. Furthermore, we need to look into the future, and another method to determine which RPC patients (such as T1/T2, N0) should receive neoadjuvant chemoradiotherapy could be through radiomics. A recently published concept^46^, AiRGOS (Artificial Intelligence, Radiomics, Genomics, Oncopathomics and Surgiomics), proposes using radiomic analysis of preoperative CT scans or MRI to help identify which patients would benefit from neoadjuvant and/or adjuvant treatments. Ultimately, this platform (AiRGOS) could also integrate other things such as pathomic, genomics and surgomic data to an algorithm to yield truly personalized medicine via an essentially AI-powered tumor board.

## Conclusions

Our NMA demonstrated that neoadjuvant chemoradiotherapy followed by surgery is the optimal choice for treating patients with RPC and BRPC. Nevertheless, it is worth noting that the current best available evidence does not encompass multiagent contemporary chemotherapy regimens. Therefore, more definitive conclusions will rely on the results of future RCTs. Further research is essential to establish stratification to improve OS and minimize treatment-related adverse events. Additionally, radiomics, as proposed by AiRGOS, offers a promising avenue to determine the suitability of neoadjuvant chemoradiation for patients. Ultimately, we anticipate close collaboration among multidisciplinary teams to bring about greater benefits for a wider range of cancer patients, including those with pancreatic cancer.

## Ethical approval

Due to the retrospective and anonymous nature of data, ethical approval was deemed not necessary.

## Consent

Not applicable.

## Financial support and sponsorship

This article was funded by the Natural Science Foundation of Liaoning Province (2021-MS-181 to CW).

## Author contribution

J.X.H., N.L., Z.Y.Y.: protocol/project development. W.Z., J.X.H., Y.X.L.: data collection or management. N.L., Z.Y.Y.: data analysis. J.X.H.: manuscript writing/editing.

## Conflicts of interest

Not applicable.

## Research registration unique identifying number (UIN)

1. Name of the registry: PROSPERO.
2. Unique Identifying number or registration ID: Comparing upfront surgery with neoadjuvant treatment in patients with resectable, borderline resectable, or locally advanced pancreatic cancer: an update network meta-analysis of randomized clinical trials (CRD42023409228).
3. Hyperlink to your specific registration (must be publicly accessible and will be checked): https://www.crd.york.ac.uk/PROSPERO/display_record.php?RecordID=409228.

## Guarantor

The corresponding author (C.L.W.) accepted full responsibility for the work and/or the conduct of the study, had access to the data, and controlled the decision to publish.

## Data availability statement

Not applicable, please contact author for data requests.

## Provenance and peer review

Not commissioned, externally peer-reviewed.

## Presentation

Not applicable.

## Supplementary Material

## References

[1] Siegel RL Miller KD Jemal A. Cancer statistics, 2019. CA Cancer J Clin 2019;69:7–34. 30620402 10.3322/caac.21551

[2] Tempero MA Malafa MP Al-Hawary M. Pancreatic Adenocarcinoma, Version 2.2021, NCCN Clinical Practice Guidelines in Oncology. J Natl Compr Cancer Network 2021;19:439–457. 10.6004/jnccn.2021.0017 33845462

[3] Khorana AA Mangu PB Berlin J. Potentially curable pancreatic cancer: American Society of Clinical Oncology Clinical Practice Guideline Update. J Clin Oncol 2017;35:2324–2328. 28398845 10.1200/JCO.2017.72.4948

[4] Groot VP Rezaee N Wu W. Patterns, timing, and predictors of recurrence following pancreatectomy for pancreatic ductal adenocarcinoma. Ann Surg 2018;267:936–945. 28338509 10.1097/SLA.0000000000002234

[5] Parikh AA Maiga A Bentrem D. Adjuvant therapy in pancreas cancer: does it influence patterns of recurrence? J Am Coll Surg 2016;222:448–456. 26895735 10.1016/j.jamcollsurg.2015.12.031 PMC10191770

[6] Suenaga M Fujii T Kanda M. Pattern of first recurrent lesions in pancreatic cancer: hepatic relapse is associated with dismal prognosis and portal vein invasion. Hepatogastroenterology 2014;61:1756–1761. 25436375

[7] Wolfgang CL Herman JM Laheru DA. Recent progress in pancreatic cancer. CA Cancer J Clin 2013;63:318–348. 23856911 10.3322/caac.21190 PMC3769458

[8] Smeenk HG Tran TC Erdmann J. Survival after surgical management of pancreatic adenocarcinoma: does curative and radical surgery truly exist? Langenbeck’s Arch Surg 2005;390:94–103. 15578211 10.1007/s00423-004-0476-9

[9] Chiaravalli M Reni M O’Reilly EM. Pancreatic ductal adenocarcinoma: State-of-the-art 2017 and new therapeutic strategies. Cancer Treat Rev 2017;60:32–43. 28869888 10.1016/j.ctrv.2017.08.007

[10] Laurence JM Tran PD Morarji K. A systematic review and meta-analysis of survival and surgical outcomes following neoadjuvant chemoradiotherapy for pancreatic cancer. J Gastrointest Surg 2011;15:2059–2069. 21913045 10.1007/s11605-011-1659-7

[11] Versteijne E Vogel JA Besselink MG. Meta-analysis comparing upfront surgery with neoadjuvant treatment in patients with resectable or borderline resectable pancreatic cancer. Br J Surg 2018;105:946–958. 29708592 10.1002/bjs.10870 PMC6033157

[12] Mokdad AA Minter RM Zhu H. Neoadjuvant therapy followed by resection versus upfront resection for resectable pancreatic cancer: a propensity score matched analysis. J Clin Oncol 2017;35:515–522. 27621388 10.1200/JCO.2016.68.5081

[13] Jang JY Han Y Lee H. Oncological benefits of neoadjuvant chemoradiation with gemcitabine versus upfront surgery in patients with borderline resectable pancreatic cancer: a prospective, randomized, open-label, multicenter phase 2/3 trial. Ann Surg 2018;268:215–222. 29462005 10.1097/SLA.0000000000002705

[14] Reni M Balzano G Zanon S. Safety and efficacy of preoperative or postoperative chemotherapy for resectable pancreatic adenocarcinoma (PACT-15): a randomised, open-label, phase 2-3 trial. The lancet. Gastroenterol Hepatol (N Y) 2018;3:413–423. 10.1016/S2468-1253(18)30081-5 29625841

[15] Cloyd JM Heh V Pawlik TM. Neoadjuvant therapy for resectable and borderline resectable pancreatic cancer: a meta-analysis of randomized controlled trials. J Clin Med 2020;9:1129. 32326559 10.3390/jcm9041129 PMC7231310

[16] van Dam JL Janssen QP Besselink MG. Neoadjuvant therapy or upfront surgery for resectable and borderline resectable pancreatic cancer: a meta-analysis of randomised controlled trials. Eur J Cancer 2022;160:140–149. 34838371 10.1016/j.ejca.2021.10.023

[17] Hajibandeh S Hajibandeh S Intrator C. Neoadjuvant chemoradiotherapy versus immediate surgery for resectable and borderline resectable pancreatic cancer: Meta-analysis and trial sequential analysis of randomized controlled trials. Ann Hepatobiliary Pancreat Surg 2023;27:28–39. 36536501 10.14701/ahbps.22-052 PMC9947376

[18] Jung HS Kim HS Kang JS. Oncologic benefits of neoadjuvant treatment versus upfront surgery in borderline resectable pancreatic cancer: a systematic review and meta-analysis. Cancers 2022;14:4360. 36139520 10.3390/cancers14184360 PMC9497278

[19] Page MJ McKenzie JE Bossuyt PM. The PRISMA 2020 statement: An updated guideline for reporting systematic reviews. Int J Surg 2021;88:105906. 33789826 10.1016/j.ijsu.2021.105906

[20] Shea BJ Reeves BC Wells G. AMSTAR 2: a critical appraisal tool for systematic reviews that include randomised or non-randomised studies of healthcare interventions, or both. BMJ (Clinical research ed) 2017;358:j4008. 10.1136/bmj.j4008 PMC5833365 28935701

[21] Abrams RA Lowy AM O’Reilly EM. Combined modality treatment of resectable and borderline resectable pancreas cancer: expert consensus statement. Ann Surg Oncol 2009;16:1751–1756. 19390900 10.1245/s10434-009-0413-9

[22] Choti MA Dixon E Tyler D. Pretreatment assessment of resectable and borderline resectable pancreatic cancer: expert consensus statement by Callery et al. Ann Surg Oncol 2009;16:1734–1735. 19390901 10.1245/s10434-009-0411-y

[23] Sterne JAC Savović J Page MJ. RoB 2: a revised tool for assessing risk of bias in randomised trials. BMJ (Clinical research ed) 2019;366:l4898. 10.1136/bmj.l4898 31462531

[24] Tierney JF Stewart LA Ghersi D. Practical methods for incorporating summary time-to-event data into meta-analysis. Trials 2007;8:16. 17555582 10.1186/1745-6215-8-16 PMC1920534

[25] Higgins JP Thompson SG Deeks JJ. Measuring inconsistency in meta-analyses. BMJ (Clinical research ed) 2003;327:557–560. 10.1136/bmj.327.7414.557 PMC192859 12958120

[26] Neupane B Richer D Bonner AJ. Network meta-analysis using R: a review of currently available automated packages. PLoS One 2014;9:e115065. 25541687 10.1371/journal.pone.0115065 PMC4277278

[27] Brooks S Gelman A. General methods for monitoring convergence of iterative simulations. J Comput Graphi Stat 1998;7:434–455.

[28] Salanti G Ades AE Ioannidis JPA. Graphical methods and numerical summaries for presenting results from multiple-treatment meta-analysis: an overview and tutorial. J Clin Epidemiol 2011;64:163–171. 20688472 10.1016/j.jclinepi.2010.03.016

[29] Casadei R Di Marco M Ricci C. Neoadjuvant chemoradiotherapy and surgery versus surgery alone in resectable pancreatic cancer: a single-center prospective, randomized, controlled trial which failed to achieve accrual targets. J Gastrointest Surg 2015;19:1802–1812. 26224039 10.1007/s11605-015-2890-4

[30] Ettrich TJ Uhl W Kornmann M. Perioperative or adjuvant nab-paclitaxel plus gemcitabine for resectable pancreatic cancer: Updated final results of the randomized phase II AIO-NEONAX trial. JCO 2022;40:4133.

[31] Ghaneh P Palmer D Cicconi S. Immediate surgery compared with short-course neoadjuvant gemcitabine plus capecitabine, FOLFIRINOX, or chemoradiotherapy in patients with borderline resectable pancreatic cancer (ESPAC5): a four-arm, multicentre, randomised, phase 2 trial. Lancet Gastroenterol Hepatol 2023;8:157–168. 36521500 10.1016/S2468-1253(22)00348-X

[32] Golcher H Brunner TB Witzigmann H. Neoadjuvant chemoradiation therapy with gemcitabine/cisplatin and surgery versus immediate surgery in resectable pancreatic cancer: results of the first prospective randomized phase II trial. Strahlentherapie Onkologie 2015;191:7–16. 10.1007/s00066-014-0737-7 PMC4289008 25252602

[33] Katz MHG Shi Q Meyers J. Efficacy of preoperative mFOLFIRINOX vs. mFOLFIRINOX plus hypofractionated radiotherapy for borderline resectable adenocarcinoma of the pancreas: The A021501 Phase 2 Randomized Clinical Trial. JAMA Oncol 2022;8:1263–1270. 35834226 10.1001/jamaoncol.2022.2319 PMC9284408

[34] Loehrer PJ Sr Feng Y Cardenes H. Gemcitabine alone versus gemcitabine plus radiotherapy in patients with locally advanced pancreatic cancer: an Eastern Cooperative Oncology Group trial. J Clin Oncol 2011;29:4105–4112. 21969502 10.1200/JCO.2011.34.8904 PMC3525836

[35] Versteijne E Suker M Groothuis K. Preoperative chemoradiotherapy versus immediate surgery for resectable and borderline resectable pancreatic cancer: results of the Dutch randomized phase III PREOPANC trial. J Clin Oncol 2020;38:1763–1773. 32105518 10.1200/JCO.19.02274 PMC8265386

[36] Uson Junior PLS Dias ESD de Castro NM. Does neoadjuvant treatment in resectable pancreatic cancer improve overall survival? A systematic review and meta-analysis of randomized controlled trials. ESMO Open 2023;8:100771. 36638709 10.1016/j.esmoop.2022.100771 PMC10024142

[37] Del Chiaro M Rangelova E Halimi A. Pancreatectomy with arterial resection is superior to palliation in patients with borderline resectable or locally advanced pancreatic cancer. HPB 2019;21:219–225. 30093144 10.1016/j.hpb.2018.07.017

[38] Mougalian SS Soulos PR Killelea BK. Use of neoadjuvant chemotherapy for patients with stage I to III breast cancer in the United States. Cancer 2015;121:2544–2552. 25902916 10.1002/cncr.29348

[39] Hu Q Wang D Chen Y. Network meta-analysis comparing neoadjuvant chemoradiation, neoadjuvant chemotherapy and upfront surgery in patients with resectable, borderline resectable, and locally advanced pancreatic ductal adenocarcinoma. Radiat Oncol 2019;14:120. 31291998 10.1186/s13014-019-1330-0 PMC6617703

[40] Xiang M Heestand GM Chang DT. Neoadjuvant treatment strategies for resectable pancreas cancer: a propensity-matched analysis of the National Cancer Database. Radiother Oncol 2020;143:101–107. 32044168 10.1016/j.radonc.2020.01.007

[41] Fietkau R Ghadimi M Grützmann R. Randomized phase III trial of induction chemotherapy followed by chemo radiotherapy or chemotherapy alone for nonresectable locally advanced pancreatic cancer: first results of the CONKO-007 trial. JCO, 40:4008. 10.1200/JCO.2022.40.16_suppl.4008

[42] Bahadoer RR Dijkstra EA van Etten B. Short-course radiotherapy followed by chemotherapy before total mesorectal excision (TME) versus preoperative chemoradiotherapy, TME, and optional adjuvant chemotherapy in locally advanced rectal cancer (RAPIDO): a randomised, open-label, phase 3 trial. Lancet Oncol 2021;22:29–42. 33301740 10.1016/S1470-2045(20)30555-6

[43] Jin J Tang Y Hu C. Multicenter, randomized, phase III trial of short-term radiotherapy plus chemotherapy versus long-term chemoradiotherapy in locally advanced rectal cancer (STELLAR). J Clin Oncol 2022;40:1681–1692. 35263150 10.1200/JCO.21.01667 PMC9113208

[44] Lin T Reddy A Hill C. The timing of surgery following stereotactic body radiation therapy impacts local control for borderline resectable or locally advanced pancreatic cancer. Cancers 2023;15:1252. 36831594 10.3390/cancers15041252 PMC9954439

[45] Doppenberg D van Dam JL Han Y. Predictive value of baseline serum carbohydrate antigen 19-9 level on treatment effect of neoadjuvant chemoradiotherapy in patients with resectable and borderline resectable pancreatic cancer in two randomized trials. Br J Surg 2023;110:1374–1380. 37440421 10.1093/bjs/znad210 PMC10480034

[46] Andrew A G Roland C Mohammed A-H. Surgomics and the Artificial intelligence, Radiomics, Genomics, Oncopathomics and Surgomics (AiRGOS) Project. Artific Intelligence Surg 2023;3:180–185.

