# Supplementary figures and images for: Comparing upfront surgery with neoadjuvant treatments in patients with resectable, borderline resectable or locally advanced pancreatic cancer: a systematic review and network meta-analysis of randomized clinical trials
Source: Int J Surg. 2024 Mar 18;110(6):3900–9. doi: 10.1097/JS9.0000000000001313 (PMC11175811; doi:10.1097/JS9.0000000000001313)

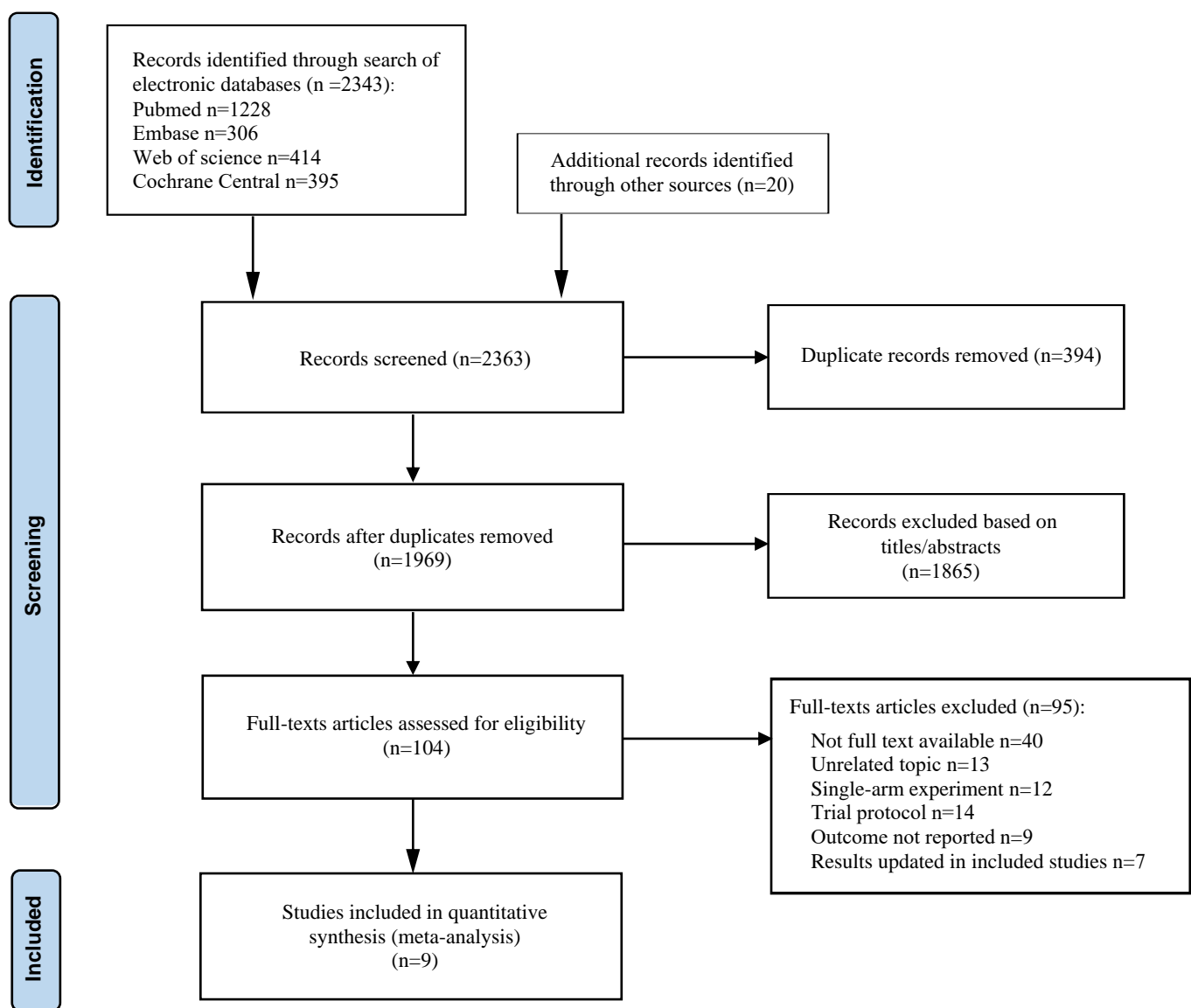

Fig. 1 Flow diagram of literature search and selection process

Supplement: Supplementary file 2 [file js9-110-3900-s002.pdf]

## As percentage (intention-to-treat)

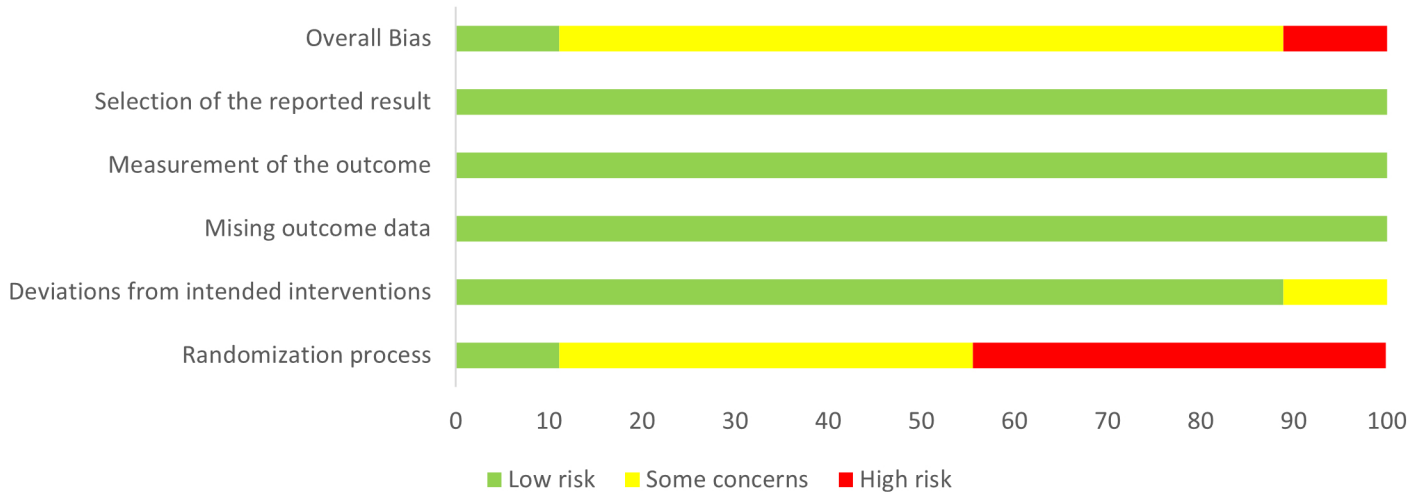

Supplement: Supplementary file 5 [file js9-110-3900-s005.pdf]
