# Supplementary material for: Comparing upfront surgery with neoadjuvant treatments in patients with resectable, borderline resectable or locally advanced pancreatic cancer: a systematic review and network meta-analysis of randomized clinical trials
Source: Int J Surg. 2024 Mar 18;110(6):3900–9. doi: 10.1097/JS9.0000000000001313 (PMC11175811; doi:10.1097/JS9.0000000000001313)
Supplement: Supplementary file 4 [file js9-110-3900-s004.docx]

**Pubmed1228**

(((((((((((((((((((((("Pancreatic Neoplasms"[Mesh]) OR (Neoplasm, Pancreatic[Title/Abstract])) OR (Pancreatic Neoplasm[Title/Abstract])) OR (Pancreas Neoplasms[Title/Abstract])) OR (Neoplasm, Pancreas[Title/Abstract])) OR (Neoplasms, Pancreas[Title/Abstract])) OR (Pancreas Neoplasm[Title/Abstract])) OR (Neoplasms, Pancreatic[Title/Abstract])) OR (Cancer of Pancreas[Title/Abstract])) OR (Pancreas Cancers[Title/Abstract])) OR (Pancreas Cancer[Title/Abstract])) OR (Cancer, Pancreas[Title/Abstract])) OR (Cancers, Pancreas[Title/Abstract])) OR (Pancreatic Cancer[Title/Abstract])) OR (Cancer, Pancreatic[Title/Abstract])) OR (Cancers, Pancreatic[Title/Abstract])) OR (Pancreatic Cancers[Title/Abstract])) OR (Cancer of the Pancreas[Title/Abstract]))) OR (pancreatic ductal adenocarcinoma[Title/Abstract])) OR (PDAC[Title/Abstract])

AND (((((((((((((((((((((((((((((((((((((((((((((((("Neoadjuvant Therapy"[Mesh]) OR (Neoadjuvant Therapies[Title/Abstract])) OR (Therapy, Neoadjuvant[Title/Abstract])) OR (Neoadjuvant Treatment[Title/Abstract])) OR (Neoadjuvant Treatments[Title/Abstract])) OR (Treatment, Neoadjuvant[Title/Abstract])) OR (Neoadjuvant Chemoradiotherapy[Title/Abstract])) OR (Chemoradiotherapy, Neoadjuvant[Title/Abstract])) OR (Neoadjuvant Chemoradiotherapies[Title/Abstract])) OR (Neoadjuvant Chemoradiation Therapy[Title/Abstract])) OR (Chemoradiation Therapy, Neoadjuvant[Title/Abstract])) OR (Neoadjuvant Chemoradiation Therapies[Title/Abstract])) OR (Therapy, Neoadjuvant Chemoradiation[Title/Abstract])) OR (Neoadjuvant Chemoradiation Treatment[Title/Abstract])) OR (Chemoradiation Treatment, Neoadjuvant[Title/Abstract])) OR (Neoadjuvant Chemoradiation Treatments[Title/Abstract])) OR (Treatment, Neoadjuvant Chemoradiation[Title/Abstract])) OR (Neoadjuvant Chemoradiation[Title/Abstract])) OR (Chemoradiation, Neoadjuvant[Title/Abstract])) OR (Neoadjuvant Chemoradiations[Title/Abstract])) OR (Neoadjuvant Radiotherapy[Title/Abstract])) OR (Neoadjuvant Radiotherapies[Title/Abstract])) OR (Radiotherapy, Neoadjuvant[Title/Abstract])) OR (Neoadjuvant Radiation Treatment[Title/Abstract])) OR (Neoadjuvant Radiation Treatments[Title/Abstract])) OR (Radiation Treatment, Neoadjuvant[Title/Abstract])) OR (Treatment, Neoadjuvant Radiation[Title/Abstract])) OR (Neoadjuvant Radiation Therapy[Title/Abstract])) OR (Neoadjuvant Radiation Therapies[Title/Abstract])) OR (Radiation Therapy, Neoadjuvant[Title/Abstract])) OR (Therapy, Neoadjuvant Radiation[Title/Abstract])) OR (Neoadjuvant Radiation[Title/Abstract])) OR (Neoadjuvant Radiations[Title/Abstract])) OR (Radiation, Neoadjuvant[Title/Abstract])) OR (Neoadjuvant Chemotherapy[Title/Abstract])) OR (Chemotherapy, Neoadjuvant[Title/Abstract])) OR (Neoadjuvant Chemotherapies[Title/Abstract])) OR (Neoadjuvant Chemotherapy Treatment[Title/Abstract])) OR (Chemotherapy Treatment, Neoadjuvant[Title/Abstract])) OR (Neoadjuvant Chemotherapy Treatments[Title/Abstract])) OR (Treatment, Neoadjuvant Chemotherapy[Title/Abstract])) OR (Neoadjuvant Systemic Therapy[Title/Abstract])) OR (Neoadjuvant Systemic Therapies[Title/Abstract])) OR (Systemic Therapy, Neoadjuvant[Title/Abstract])) OR (Therapy, Neoadjuvant Systemic[Title/Abstract])) OR (Neoadjuvant Systemic Treatment[Title/Abstract])) OR (Neoadjuvant Systemic Treatments[Title/Abstract])) OR (Systemic Treatment, Neoadjuvant[Title/Abstract])) OR (Treatment, Neoadjuvant Systemic[Title/Abstract])

AND ("randomized controlled trial"[pt] OR "controlled clinical trial"[pt] OR randomized[tiab] OR placebo[tiab] OR "drug therapy"[sh] OR randomly[tiab] OR trial[tiab] OR groups[tiab]))

AND (2009:2023[pdat])

**Web of science414**

**#1 TS=(Pancreatic Neoplasms OR Neoplasm, Pancreatic OR Pancreatic Neoplasm OR Pancreas Neoplasms OR Neoplasm, Pancreas OR Neoplasms, Pancreas OR Pancreas Neoplasm OR Neoplasms, Pancreatic OR Cancer of Pancreas OR Pancreas Cancers OR Pancreas Cancer OR Cancer, Pancreas OR Cancers, Pancreas OR Pancreatic Cancer OR Cancer, Pancreatic OR Cancers, Pancreatic OR Pancreatic Cancers OR Cancer of the Pancreas OR pancreatic ductal adenocarcinoma OR PDAC)**

**#2** TS=(Neoadjuvant Therapies OR Therapy, Neoadjuvant OR Neoadjuvant Treatment OR Neoadjuvant Treatments OR Treatment, Neoadjuvant OR Neoadjuvant Chemoradiotherapy OR Chemoradiotherapy, Neoadjuvant OR Neoadjuvant Chemoradiotherapies OR Neoadjuvant Chemoradiation Therapy OR Chemoradiation Therapy, Neoadjuvant OR Neoadjuvant Chemoradiation Therapies OR Therapy, Neoadjuvant Chemoradiation OR Neoadjuvant Chemoradiation Treatment OR Chemoradiation Treatment, Neoadjuvant OR Neoadjuvant Chemoradiation Treatments OR Treatment, Neoadjuvant Chemoradiation OR Neoadjuvant Chemoradiation OR Chemoradiation, Neoadjuvant OR Neoadjuvant Chemoradiations OR Neoadjuvant Radiotherapy OR Neoadjuvant Radiotherapies OR Radiotherapy, Neoadjuvant OR Neoadjuvant Radiation Treatment OR Neoadjuvant Radiation Treatments OR Radiation Treatment, Neoadjuvant OR Treatment, Neoadjuvant Radiation OR Neoadjuvant Radiation Therapy OR Neoadjuvant Radiation Therapies OR Radiation Therapy, Neoadjuvant OR Therapy, Neoadjuvant Radiation OR Neoadjuvant Radiation OR Neoadjuvant Radiations OR Radiation, Neoadjuvant OR Neoadjuvant Chemotherapy OR Chemotherapy, Neoadjuvant OR Neoadjuvant Chemotherapies OR Neoadjuvant Chemotherapy Treatment OR Chemotherapy Treatment, Neoadjuvant OR Neoadjuvant Chemotherapy Treatments OR Treatment, Neoadjuvant Chemotherapy OR Neoadjuvant Systemic Therapy OR Neoadjuvant Systemic Therapies OR Systemic Therapy, Neoadjuvant OR Therapy, Neoadjuvant Systemic OR Neoadjuvant Systemic Treatment OR Neoadjuvant Systemic Treatments OR Systemic Treatment, Neoadjuvant OR Treatment, Neoadjuvant Systemic)

**#3 TS= (Randomized controlled OR Randomized OR Placebo OR RCT)**

**#4 #1 AND #3 AND #4**

**#5 #4** and 2022 or 2021 or 2020 or 2019 or 2018 or 2017 or 2016 or 2015 or 2014 or 2013 or 2012 or 2011 or 2010 or 2009

**Embase306**

#1 pancreatic AND neoplasms

#2 'neoplasm, pancreatic':ab,ti OR 'pancreatic neoplasm':ab,ti OR 'pancreas neoplasms':ab,ti OR 'neoplasm, pancreas':ab,ti OR 'neoplasms, pancreas':ab,ti OR 'pancreas neoplasm':ab,ti OR 'neoplasms, pancreatic':ab,ti OR 'cancer of pancreas':ab,ti OR 'pancreas cancers':ab,ti OR 'pancreas cancer':ab,ti OR 'cancer, pancreas':ab,ti OR 'cancers, pancreas':ab,ti OR 'pancreatic cancer':ab,ti OR 'cancer, pancreatic':ab,ti OR 'cancers, pancreatic':ab,ti OR 'pancreatic cancers':ab,ti OR 'cancer of the pancreas':ab,ti OR 'pancreatic ductal adenocarcinoma':ab,ti OR 'pdac':ab,ti

#3 #1 AND #2

#4 neoadjuvant AND therapies

#5 'therapy, neoadjuvant':ab,ti OR 'neoadjuvant treatment':ab,ti OR 'neoadjuvant treatments':ab,ti OR 'treatment, neoadjuvant':ab,ti OR 'neoadjuvant chemoradiotherapy':ab,ti OR 'chemoradiotherapy, neoadjuvant':ab,ti OR 'neoadjuvant chemoradiotherapies':ab,ti OR 'neoadjuvant chemoradiation therapy':ab,ti OR 'chemoradiation therapy, neoadjuvant':ab,ti OR 'neoadjuvant chemoradiation therapies':ab,ti OR 'therapy, neoadjuvant chemoradiation':ab,ti OR 'neoadjuvant chemoradiation treatment':ab,ti OR 'chemoradiation treatment, neoadjuvant':ab,ti OR 'neoadjuvant chemoradiation treatments':ab,ti OR 'treatment, neoadjuvant chemoradiation':ab,ti OR 'neoadjuvant chemoradiation':ab,ti OR 'chemoradiation, neoadjuvant':ab,ti OR 'neoadjuvant chemoradiations':ab,ti OR 'neoadjuvant radiotherapy':ab,ti OR 'neoadjuvant radiotherapies':ab,ti OR 'radiotherapy, neoadjuvant':ab,ti OR 'neoadjuvant radiation treatment':ab,ti OR 'neoadjuvant radiation treatments':ab,ti OR 'radiation treatment, neoadjuvant':ab,ti OR 'treatment, neoadjuvant radiation':ab,ti OR 'neoadjuvant radiation therapy':ab,ti OR 'neoadjuvant radiation therapies':ab,ti OR 'radiation therapy, neoadjuvant':ab,ti OR 'therapy, neoadjuvant radiation':ab,ti OR 'neoadjuvant radiation':ab,ti OR 'neoadjuvant radiations':ab,ti OR 'radiation, neoadjuvant':ab,ti OR 'neoadjuvant chemotherapy':ab,ti OR 'chemotherapy, neoadjuvant':ab,ti OR 'neoadjuvant chemotherapies':ab,ti OR 'neoadjuvant chemotherapy treatment':ab,ti OR 'chemotherapy treatment, neoadjuvant':ab,ti OR 'neoadjuvant chemotherapy treatments':ab,ti OR 'treatment, neoadjuvant chemotherapy':ab,ti OR 'neoadjuvant systemic therapy':ab,ti OR 'neoadjuvant systemic therapies':ab,ti OR 'systemic therapy, neoadjuvant':ab,ti OR 'therapy, neoadjuvant systemic':ab,ti OR 'neoadjuvant systemic treatment':ab,ti OR 'neoadjuvant systemic treatments':ab,ti OR 'systemic treatment, neoadjuvant':ab,ti OR 'treatment, neoadjuvant systemic':ab,ti

#6 #4 AND #5

#7 ('randomized controlled':ab,ti OR 'randomized':ab,ti OR 'placebo':ab,ti OR 'rct':ab,ti)

#8 #3 AND #6 AND #7

#9 #8 AND [2009-2023]/py

**Cochrane 395**

#1 Pancreatic Neoplasms

#2 (Neoplasm, Pancreatic):ab,ti,kw OR (Pancreatic Neoplasm):ab,ti,kw OR (Pancreas Neoplasms):ab,ti,kw OR (Neoplasm, Pancreas):ab,ti,kw OR (Neoplasms, Pancreas):ab,ti,kw OR (Pancreas Neoplasm):ab,ti,kw OR (Neoplasms, Pancreatic):ab,ti,kw OR (Cancer of Pancreas):ab,ti,kw OR (Pancreas Cancers):ab,ti,kw OR (Pancreas Cancer):ab,ti,kw OR (Cancer, Pancreas):ab,ti,kw OR (Cancers, Pancreas):ab,ti,kw OR (Pancreatic Cancer):ab,ti,kw OR (Cancer, Pancreatic):ab,ti,kw OR (Cancers, Pancreatic):ab,ti,kw OR (Pancreatic Cancers):ab,ti,kw OR (Cancer of the Pancreas):ab,ti,kw OR (pancreatic ductal adenocarcinoma):ab,ti,kw OR (PDAC):ab,ti,kw

#3 Neoadjuvant Therapy

#4 (Neoadjuvant Therapies):ab,ti,kw OR (Therapy, Neoadjuvant):ab,ti,kw OR (Neoadjuvant Treatment):ab,ti,kw OR (Neoadjuvant Treatments):ab,ti,kw OR (Treatment, Neoadjuvant):ab,ti,kw OR (Neoadjuvant Chemoradiotherapy):ab,ti,kw OR (Chemoradiotherapy, Neoadjuvant):ab,ti,kw OR (Neoadjuvant Chemoradiotherapies):ab,ti,kw OR (Neoadjuvant Chemoradiation Therapy):ab,ti,kw OR (Chemoradiation Therapy, Neoadjuvant):ab,ti,kw OR (Neoadjuvant Chemoradiation Therapies):ab,ti,kw OR (Therapy, Neoadjuvant Chemoradiation):ab,ti,kw OR (Neoadjuvant Chemoradiation Treatment):ab,ti,kw OR (Chemoradiation Treatment, Neoadjuvant):ab,ti,kw OR (Neoadjuvant Chemoradiation Treatments):ab,ti,kw OR (Treatment, Neoadjuvant Chemoradiation):ab,ti,kw OR (Neoadjuvant Chemoradiation):ab,ti,kw OR (Chemoradiation, Neoadjuvant):ab,ti,kw OR (Neoadjuvant Chemoradiations):ab,ti,kw OR (Neoadjuvant Radiotherapy):ab,ti,kw OR (Neoadjuvant Radiotherapies):ab,ti,kw OR (Radiotherapy, Neoadjuvant):ab,ti,kw OR (Neoadjuvant Radiation Treatment):ab,ti,kw OR (Neoadjuvant Radiation Treatments):ab,ti,kw OR (Radiation Treatment, Neoadjuvant):ab,ti,kw OR (Treatment, Neoadjuvant Radiation):ab,ti,kw OR (Neoadjuvant Radiation Therapy):ab,ti,kw OR (Neoadjuvant Radiation Therapies):ab,ti,kw OR (Radiation Therapy, Neoadjuvant):ab,ti,kw OR (Therapy, Neoadjuvant Radiation):ab,ti,kw OR (Neoadjuvant Radiation):ab,ti,kw OR (Neoadjuvant Radiations):ab,ti,kw OR (Radiation, Neoadjuvant):ab,ti,kw OR (Neoadjuvant Chemotherapy):ab,ti,kw OR (Chemotherapy, Neoadjuvant):ab,ti,kw OR (Neoadjuvant Chemotherapies):ab,ti,kw OR (Neoadjuvant Chemotherapy Treatment):ab,ti,kw OR (Chemotherapy Treatment, Neoadjuvant):ab,ti,kw OR(Neoadjuvant Chemotherapy Treatments):ab,ti,kw OR(Treatment, Neoadjuvant Chemotherapy):ab,ti,kw OR(Neoadjuvant Systemic Therapy):ab,ti,kw OR(Neoadjuvant Systemic Therapies):ab,ti,kw OR(Systemic Therapy, Neoadjuvant):ab,ti,kw OR(Therapy, Neoadjuvant Systemic):ab,ti,kw OR(Neoadjuvant Systemic Treatment):ab,ti,kw OR(Neoadjuvant Systemic Treatments):ab,ti,kw OR(Systemic Treatment, Neoadjuvant):ab,ti,kw OR(Treatment, Neoadjuvant Systemic):ab,ti,kw

#5 #1 OR #2

#6 #3 OR #4

#7 Randomized controlled

#8 (Randomized):ab,ti,kw OR (Placebo):ab,ti,kw OR (RCT):ab,ti,kw

#9 #7 OR #8

#10 #5 AND #6 AND #9 with Cochrane Library publication date from Jan 2009 to Oct 2023
