# Supplementary material for: Comparing upfront surgery with neoadjuvant treatments in patients with resectable, borderline resectable or locally advanced pancreatic cancer: a systematic review and network meta-analysis of randomized clinical trials
Source: Int J Surg. 2024 Mar 18;110(6):3900–9. doi: 10.1097/JS9.0000000000001313 (PMC11175811; doi:10.1097/JS9.0000000000001313)
Supplement: Supplementary file 6 [file js9-110-3900-s006.pdf]

	Randomization process	Deviations from intended intervention	Missing outcome data	Measurement of the outcome	Selection of the reported result	Overall
casadei 2015	?	+	+	+	+	!
Reni 2018	+	+	+	+	+	+
Versteijne 2022	?	?	+	+	+	?
Loehrer 2011	?	+	+	+	+	!
Jang 2018	?	+	+	+	+	!
Golcher 2014	?	+	+	+	+	!
Katz 2022	?	+	+	+	+	!
Ettrich 2022	?	+	+	+	+	!
Ghaneh 2022	?	+	+	+	+	!
