# Supplementary material for: Comparing upfront surgery with neoadjuvant treatments in patients with resectable, borderline resectable or locally advanced pancreatic cancer: a systematic review and network meta-analysis of randomized clinical trials
Source: Int J Surg. 2024 Mar 18;110(6):3900–9. doi: 10.1097/JS9.0000000000001313 (PMC11175811; doi:10.1097/JS9.0000000000001313)
Supplement: Supplementary file 7 [file js9-110-3900-s007.docx]

Results (random effects model):

treat1 treat2 HR 95%-CI

Casadei 2015 CRT US 0.7936 [0.6750; 0.9330]

Reni 2018 CT US 1.0057 [0.8586; 1.1779]

Versteijne 2022 CRT US 0.7936 [0.6750; 0.9330]

Loehrer Sr 2011 CRT CT 0.7891 [0.6364; 0.9785]

Jang 2018 CRT US 0.7936 [0.6750; 0.9330]

Golcher 2014 CRT US 0.7936 [0.6750; 0.9330]

Ettrich 2022 CT US 1.0057 [0.8586; 1.1779]

Ghaneh 2022(1) CT US 1.0057 [0.8586; 1.1779]

Ghaneh 2022(2) CRT US 0.7936 [0.6750; 0.9330]

Ghaneh 2022(3) CRT CT 0.7891 [0.6364; 0.9785]

Number of studies: k = 10

Number of treatments: n = 3

Number of pairwise comparisons: m = 10

Number of designs: d = 1

Random effects model

Treatment estimate (HR):

CRT CT US

CRT . 0.7891 0.7936

CT 1.2672 . 1.0057

US 1.2601 0.9944 .

Lower 95%-confidence limit:

CRT CT US

CRT . 0.6364 0.6750

CT 1.0219 . 0.8586

US 1.0718 0.8490 .

Upper 95%-confidence limit:

CRT CT US

CRT . 0.9785 0.9330

CT 1.5714 . 1.1779

US 1.4815 1.1647 .

Quantifying heterogeneity:

tau^2 = 0; tau = 0; I^2 = 0% [0.0%; 64.8%]

Test of heterogeneity:

Q d.f. p-value

4.76 8 0.7826

Q statistics to assess homogeneity / consistency

Q df p-value

Total 4.76 8 0.7826

Within designs 4.73 7 0.6925

Between designs 0.03 1 0.8636

Design-specific decomposition of within-designs Q statistic

Design Q df p-value

CRT:CT 0.25 1 0.6182

CRT:US 1.56 4 0.8160

CT:US 2.93 2 0.2316

Between-designs Q statistic after detaching of single designs

Detached design Q df p-value

CRT:CT 0.00 0 --

CRT:US 0.00 0 --

CT:US 0.00 0 --

Q statistic to assess consistency under the assumption of

a full design-by-treatment interaction random effects model

Q df p-value tau.within tau2.within

Between designs 0.03 1 0.8636 0 0

League table (random effects model):

CRT 0.80 (0.67, 0.94) 0.76 (0.45, 1.27)

0.79 (0.67, 0.93) US 1.00 (0.85, 1.18)

0.79 (0.64, 0.98) 0.99 (0.85, 1.16) CT

SUCRA

P-score

CRT 0.9910

US 0.2652

CT 0.2438

> nr2<-print(nr[3],sort=TRUE)

$ranking.random

CRT CT US

0.9909810 0.2438120 0.2652071
